# Rethinking Melatonin Dosing: Safety and Efficacy at Higher-than-Usual Levels in Aged Patients with Sleep Disturbances and Comorbidities

**DOI:** 10.3390/brainsci15101040

**Published:** 2025-09-25

**Authors:** Stella M. Valiensi, Vanesa A. Vera, Agustín L. Folgueira, Sofía Caporale, Marcela Ponce de León, Isis Pino Fernández, Daniel E. Vigo, Daniel P. Cardinali

**Affiliations:** 1 Neurología-Medicina del Sueño, Hospital Italiano de Buenos Aires, Buenos Aires C1199ABB, Argentina; stellamaris.valiensi@hospitalitaliano.org.ar (S.M.V.); antonella.vera@hospitalitaliano.org.ar (V.A.V.); agustin.folgueira@hospitalitaliano.org.ar (A.L.F.); sofia.caporale@hospitalitaliano.org.ar (S.C.); marcela.ponce@hospitalitaliano.org.ar (M.P.d.L.); isis.pino@hospitalitaliano.org.ar (I.P.F.); 2 Institute for Biomedical Research (BIOMED), National Scientific and Technical Research Council and Pontificia Universidad Católica Argentina, Buenos Aires C1107AAZ, Argentina; danielvigo@uca.edu.ar; 3 Facultad de Ciencias Médicas, Pontificia Universidad Católica Argentina, Buenos Aires C1107AAZ, Argentina

**Keywords:** arterial hypertension, chronobiotic, cytoprotection, diabetes mellitus, high melatonin dose, ischemic heart disease, sleep

## Abstract

**Background**. Although melatonin is widely used in Sleep Medicine for its chronobiological action, its potent antioxidant and mitochondrial regulatory effects, as well as its immunomodulatory and anti-inflammatory functions, make it of interest as a cytoprotective agent in several chronic pathologies. These actions are evident at doses higher than those used for sleep disorders. Even at high doses, melatonin’s adverse effects are few, mild, and self-limited or resolve quickly after discontinuation of treatment. Based on its safety profile, we treated melatonin for sleep disorders in the presence of comorbidities with doses ≥ 40 mg daily. **Methods**. This was a retrospective mixed observational analytical design comprising a retrospective uncontrolled cohort analysis and a cross-sectional study. Eighty-one patients (57 female) with sleep disorders ranging in age from 55 to 98 years (mean 74.4 years) were treated with melatonin 40 to 200 mg daily (mean 72.7 mg) were examined. Fifty-six percent of patients received treatment for more than 4 years. The control group for the cross-sectional analysis included 81 patients over 52 years of age, matched by age and sex and not receiving melatonin but having sleep disorders within the same period. **Results**. A significant decrease was observed in arterial hypertension, ischemic heart disease and diabetes mellitus after melatonin administration. Analysis of clinical laboratory variables indicated no changes in the treated group versus the untreated group, except for a lower alkaline phosphatase concentration in patients who received melatonin. **Conclusions**. These findings suggest a beneficial effect of cytoprotective doses of melatonin on the cardiovascular and metabolic profile in an aged population.

## 1. Introduction

Melatonin is frequently administered in clinical settings at chronobiotic doses (typically ≤10 mg daily) [1,2,3]. However, experimental research highlights its broader potential as an antioxidant, immunological and mitochondrial regulator and anti-inflammatory agent [4,5,6,7]. Allometric scaling from animal studies suggests significantly higher doses for humans, ranging from 75 to 112.5 mg daily for a 75 kg adult [8,9]. Given that Phase 1 pharmacological studies have shown no toxicity with melatonin doses up to 100 mg in healthy volunteers [10,11], we usually employed this higher dose range to manage sleep disorders in elderly patients and potentially mitigate age-related comorbidities.

Recent studies have further explored melatonin’s role at high doses in various physiological processes. For instance, its impact on cardiovascular health has been documented, showing that melatonin can act as a chronobiotic and cytoprotective agent in the cardiovascular system (see references [4,12,13,14,15,16,17,18]). Moreover, melatonin’s antioxidant properties have been linked to its ability to protect against oxidative stress and inflammation, which are common in various chronic diseases [19]. Melatonin’s role in cancer therapy, showing its potential to enhance the efficacy of conventional treatments and reduce side effects, is relevant [20]. Additionally, melatonin has been investigated for its neuroprotective properties in neurodegenerative diseases such as Alzheimer’s and Parkinson’s [21].

In a previous study we reviewed clinical history data from aged patients who were taking melatonin at a high dose for various sleep disorders [12]. The prescribed melatonin doses varied from 40 to 200 mg per day, with an average daily dose of 77 mg. The findings indicated that liver function markers remained within the normal range in all patients, irrespective of the melatonin dose.

As a follow-up to that study, we now present data supporting a beneficial effect of a cytoprotective dose of melatonin on the cardiovascular and metabolic profile of the analyzed group. The present study had the following objectives: 1. To analyze the presence of non-communicable diseases before and after receiving high doses of melatonin. 2. To compare clinical laboratory tests in these patients with those of an age- and sex-matched control group.

## 2. Patients and Methods

### 2.1. Design

**Figure 1:**
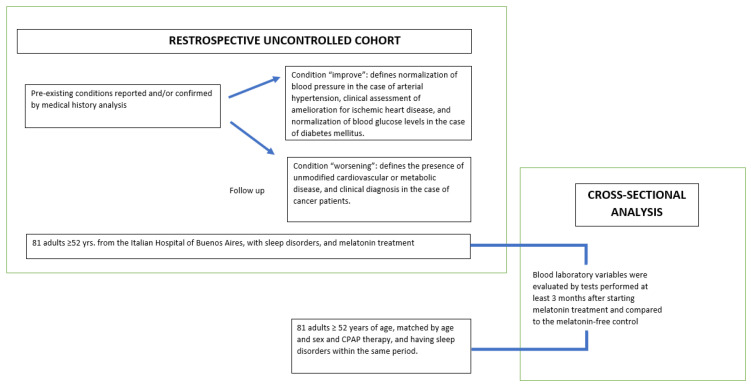
Study design illustrating the retrospective uncontrolled cohort of 81 adults with sleep disorders treated with melatonin at the Italian Hospital of Buenos Aires, where pre and post treatment assessments were used to evaluate improvement or worsening of preexisting conditions. The design also includes a cross-sectional comparison of post-treatment laboratory variables with an age, sex, and CPAP therapy matched control group.

This retrospective study employed a mixed observational analytical design comprising two components: a retrospective uncontrolled cohort analysis and a cross-sectional analysis (Figure 1). Both components examined clinical records from a defined, closed database comprising patients from the Italian Hospital of Buenos Aires. The individuals included had all been evaluated and managed for sleep-related disorders within the Sleep Medicine Section between 1 January 2013, and 31 December 2024, and the study protocol was formally registered in the Buenos Aires Registry of Research Protocols (PRISSA), entry number 15,593. Eligibility criteria involved adults aged 52 and older, covered exclusively under the hospital’s internal healthcare system.

### 2.2. Participants

The melatonin group included 81 patients that received fast-release melatonin at bedtime to treat a variety of sleep disorders such as insomnia (either due to sleep onset disorders, sleep maintenance disorders, and/or early awakening); COMISA (insomnia associated with apneas with or without CPAP treatment); Sleep parasomnias such as REM sleep behavior disorder; Circadian rhythm disorders; Hypersomnia (or a reason such as excessive sleepiness); Circadian rhythm misalignments of various etiologies.

The control group for the cross-sectional analysis included 81 patients over 52 years of age, matched by age and sex and CPAP therapy, and having sleep disorders within the same period.

Patients in both groups were followed through clinical interviews conducted at intervals of 4 to 6 months. Biochemical monitoring included baseline liver function tests and annual follow-up assessments. Prescribed treatments during follow-up could include pregabalin, gabapentin, Z-drugs, antipsychotics, or antidepressants, according to clinical indications.

### 2.3. Melatonin Administration

Administered daily doses of fast release melatonin were given p.o. at bedtime, adjusted according to the patient’s age and relevant comorbidities identified in clinical assessment. To be included in the analysis, patients had to adhere to the prescribed regimen for a minimum of 4 consecutive months. A successful therapeutic response was determined by the patient’s follow-up evaluation at 4 months and continued treatment eligibility by the patient as evidenced by prescriptions extending beyond one year.

### 2.4. Measures

Several variables related to preexisting conditions reported and/or confirmed by medical history analysis were recorded. For the cohort analysis, the condition “improve” defines normalization of blood pressure in the case of arterial hypertension, clinical assessment of amelioration for ischemic heart disease, and normalization of blood glucose levels in the case of diabetes mellitus. The condition “worsening” defines the presence of unmodified cardiovascular or metabolic disease, and clinical diagnosis in the case of cancer patients. For the cross-sectional analysis, blood laboratory variables were evaluated by tests performed at least 3 months after starting melatonin treatment and compared to the melatonin-free control group.

### 2.5. Statistical Analysis

For qualitative variables, results were expressed as frequencies and percentages. Quantitative variables were expressed as mean and standard deviation. A McNemar test was used to analyze significant changes in the condition status (improvement or worsening) after melatonin treatment. Mann–Whitney tests were used to analyze continuous variables, and Chi-square tests were used to compare categorical variables between the melatonin and control groups. All statistical analyses were performed using the Statistical Package for the Social Sciences software (version 25.0; SPSS Inc., 233 South Wacker Drive, Chicago, IL 60606, USA).

## 3. Results

A predominantly female sample (70.7%) was prescribed with melatonin doses ranging from 40 to 200 mg daily, with a mean of 72.7 ± 33.1. Duration of treatment and number of patients receiving melatonin were less than one year, 3; from one to three years, 33; from four to six years, 17; from six to ten years, 17; more than ten years, 11. In both groups, no clinically relevant adverse effects were observed; only occasional symptoms such as headache or dizziness were reported, but these were not systematically recorded.

Table 1 summarizes the comparative analysis of sociodemographic variables and medical history in melatonin-treated and age- and sex-matched control groups. Significant differences between groups were seen in liver disease (encompassing diagnoses such as hepatic steatosis, hepatitis, or cirrhosis based on clinical history), alcohol consumption, defined as ≥1 glass per day, pain/bruxism/fibromyalgia (grouped due to overlapping symptomatology and shared clinical relevance in sleep pathology) and Parkinson’s disease which was reported only in the melatonin group. It must be stressed that self-reported history variables may be subject to recall bias or underreporting particularly for lifestyle-related conditions.

A Mann–Whitney test was used to compare differences between groups for age, and chi-square tests were used to compare differences between groups for categorical variables. A *p* < 0.05 indicates statistical significance. CPAP therapy refers to current or prior use of continuous positive airway pressure therapy for sleep apnea. COPD: Chronic Obstructive Pulmonary Disease. Self-reported history variables may be subject to recall bias or underreporting, particularly for lifestyle-related conditions.

**Figure 2:**
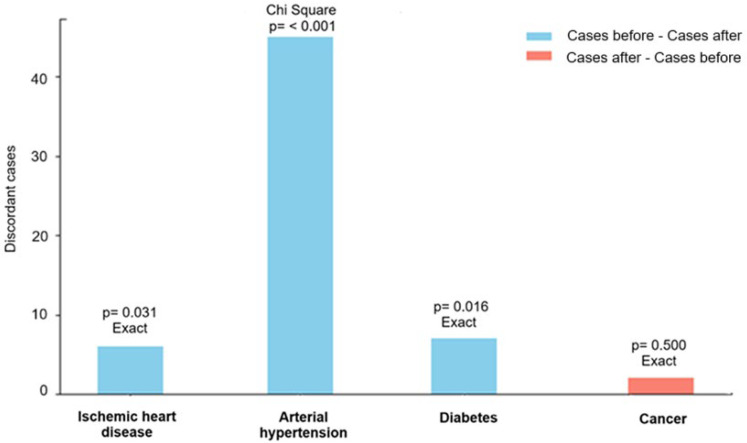
McNemar’s test allows evaluating differences in dichotomous variables in paired data of Table 2. The chi-square approximation was used for arterial hypertension (b + c = 45), and the exact test was applied for all other conditions (b + c < 25).

Table 2 and Figure 2 analyze the existence of improvements or worsening in non-communicable diseases before and after receiving high-dose melatonin. The McNemar test was applied to analyze significant changes in the prevalence of diseases before and after melatonin treatment. This statistical test allows for the evaluation of differences in dichotomous variables in paired data.

The condition “improve” defines normalization of blood pressure in the case of arterial hypertension, clinical assessment of amelioration for ischemic heart disease, and normalization of blood glucose levels in the case of diabetes mellitus. McNemar’s test was applied to assess significant changes in the number of subjects who improved or worsened their conditions before and after melatonin treatment. The chi-square approximation was used for arterial hypertension (b + c = 45), and the exact test was applied for all other conditions (b + c < 25).

A statistically significant decrease was observed in hypertension, ischemic heart disease and diabetes after melatonin administration, while changes in oncological pathologies did not reach significance. These findings indicate a beneficial effect of melatonin on the cardiovascular and metabolic profile of the analyzed group.

Table 3 shows the comparative analysis of the chemical laboratory variables of the study population taking melatonin and the control group, matched by age, sex and CPAP therapy. The control group had higher mean serum alkaline phosphatase (ALP) values than those taking melatonin, (*p* = 0.02). No other significant differences were found.

## 4. Discussion

Results obtained in this observational, analytical, and mixed design study support the conclusion that melatonin in high doses and for a relatively long period of time is associated with improvement in hypertension, ischemic heart disease and diabetes mellitus in aged individuals, although casualty cannot be inferred. Melatonin diminishes progressively with aging and is especially reduced in numerous chronic, age-related non-communicable diseases [13,14]. In humans, beyond its role in regulating circadian rhythms, melatonin demonstrates multifaceted protective functions in cardiovascular disease, involving antioxidant activity, modulation of inflammation, and epigenetic control [12].

Cardiovascular diseases are the leading cause of death and disability worldwide and increasing evidence has demonstrated the beneficial effects of melatonin in preventing and improving cardiovascular risk factors. Exogenous administration of melatonin, because of its antioxidant and anti-inflammatory properties, has been reported to decrease blood pressure, protect against atherosclerosis, attenuate molecular and cellular damage resulting from cardiac ischemia/reperfusion, and improve the prognosis of myocardial infarction and heart failure (see references [4,12,13,14,15,16,17,18]).

Atherosclerosis stands as a central contributor to the sudden death of countless individuals each year. In many cases, fatalities arise from the rupture of an unstable arterial plaque, which subsequently releases cellular remnants and biochemical material into the vessel’s interior. Melatonin plays an essential role in fortifying vulnerable plaques, promoting their transformation into more stable structures (see references [20,21,22]). Laboratory investigations indicate that melatonin limits neovascularization from the tunica media into the plaque, thereby easing mechanical stress, preventing hemorrhage within the plaque, and curbing the expansion of the necrotic zone. Furthermore, melatonin enhances collagen production by infiltrating vascular smooth muscle cells, thereby reinforcing the structural integrity of the plaque’s surface. Its notable antioxidant and anti-inflammatory properties help mitigate oxidative harm in surrounding tissues and dampen inflammatory processes—both key factors in plaque destabilization [20,21,22,23,24,25,26].

Both melatonin and the synthetic agonist piromelatine have shown potential to restore insulin signaling functionality [27]. Clinical evidence on melatonin’s role in managing diabetes mellitus has recently been reviewed in a meta-analysis of 9 randomized controlled trials involving 427 patients with type 2 diabetes mellitus [28]. Melatonin supplementation significantly reduced HbA1c levels with no significant change in fasting plasma glucose. These findings suggest that melatonin improves long-term glycemic control but not acute changes in glucose levels [27,28,29,30,31,32,33,34,35,36,37].

Melatonin also plays a defensive role by preventing biochemical cascades that drive inflammation. This includes processes such as calcium dysregulation, uncontrolled nitric oxide release, the formation of peroxynitrite and its radical derivatives, and mitochondrial impairment due to oxidative burden [33,34]. These damaging effects are associated with low-grade inflammation observed across multiple tissues, especially as part of the aging process. Experimental evidence in animal models indicates that melatonin helps combat these issues through mitochondrial stabilization and reduction in peroxynitrite-related cellular damage [35].

The immunomodulatory role of melatonin is also essential to its cytoprotective profile. It influences both immune activation and suppression, resulting in varied redox states—prooxidant or antioxidant—depending on physiological context [36,37,38]. Notably, the anti-inflammatory actions of melatonin gain relevance with advancing age.

In the present study, when analyzing the population taking melatonin and the control group, matched by age, sex and CPAP therapy, the only alteration that emerged was that the melatonin group had lower mean serum alkaline phosphatase values (ALP) than controls. Two reasons can be entertained for this finding [39]: (a) Melatonin’s dampening of bone turnover, which is typically elevated in osteoporosis, especially in postmenopausal and age-related forms of the disease [40]. Since melatonin slows osteoclast-driven resorption and modulates osteoblast activity [9,41], then the bone-specific ALP fraction in serum would drop. (b) Liver protection [42,43]. In cholestatic or toxic injury models, melatonin can reduce bile-duct stress and hepatocellular damage, which in turn lowers the biliary isoform of ALP in the blood. In the present study the coexistence of lower ALP levels with normality of other markers of liver disease, like liver enzymes (GOT, GPT) or bilirubin, tends to rule out liver involvement and suggest a decreasing bone turnover by melatonin, a beneficial effect for this predominantly female population. Further studies disclosing the type of ALP measured, as well as the evaluation of markers of bone resorption, will clarify these queries.

The present investigation faces several limitations. Given the retrospective nature of the data collection, certain pertinent data were not captured, and the fixed nature of the variables precluded any subsequent adjustments or additions. In this regard, baseline biochemical indicators were not systematically collected prior to melatonin administration, preventing comparisons with the control group. We recognize this as an important limitation of the study. Although previous research has reported positive effects of melatonin on lipid profiles—specifically on total cholesterol, LDL, and triglycerides [44,45,46,47]—these benefits were not evident in our cohort. An additional limitation is that therapeutic outcomes were assessed through subjective reports exclusively based on patient feedback. Participants noted improvements such as reduced sleep fragmentation and quicker sleep onset, and they consistently sought repeat prescriptions every 4 months, suggesting they experienced sustained enhancements in sleep efficiency. Nevertheless, the reliance on a closed database limited the availability of standardized outcome measures that could have further substantiated these reports. Another notable limitation lies in the gender imbalance, with a predominance of female participants. This could have skewed the findings, given that epidemiological data associate female sex with elevated rates of sleep disturbances as insomnia. The absence of detailed information on comorbidities and concomitant medications, and the lack of matching for these potential confounders—particularly cardiovascular and metabolic diseases—also represent important limitations of this study. In this regard, although certain comorbidities (e.g., liver disease, Parkinson’s disease, pain, bruxism, fibromyalgia) may have influenced the outcomes, we chose not to exclude these patients in order to preserve the representativeness of the sample. This constitutes a trade-off limitation, reflecting a balance between stricter internal control and broader external validity.

## 5. Conclusions

This study demonstrates that long-term administration of high-dose melatonin (40–200 mg/day) in older adults with sleep disorders is well tolerated and suggests that it is associated with improvements in arterial hypertension, ischemic heart disease, and diabetes mellitus, alongside favorable modulation of alkaline phosphatase levels without other laboratory alterations. Long-term clinical studies evaluating the safety of high-dose melatonin in elderly patients remain scarce. While our study provides long-term data supporting the safety of high-dose melatonin in elderly patients, further studies are needed to fully establish its long-term risks. The observed improvement in the studied conditions should be interpreted as correlations rather than proof of a protective effect, although they are consistent with the cytoprotective potential of supraphysiological melatonin dosing for improving cardiovascular and metabolic health in the elderly. Future prospective, long-term, randomized trials are warranted to confirm safety and efficacy as well as to define optimal dosing strategies.

## Figures and Tables

**Table 1:** Demographic variables and medical history of melatonin-treated and control groups.

Variable	Melatonin				Control				*p*
	N = 81				N = 81				
Demographic variables									
	Min	Max	Mean	SD	Min	Max	Mean	SD	
Age (years)	55	98	74.4	9.1	52	92	73.9	8.9	0.71
	N		%		N		%		
Female	57		70.7		55		67.9		0.69
Male	24		29.3		26		32.1		
Patient-reported history									
	N		%		N		%		
CPAP therapy	36		44.4		38		46.9		0.70
Alcohol consumption	2		2.4		8		9.9		0.05
Liver disease	4		4.9		0		0		0.04
Arterial hypertension	54		65.9		45		55.6		0.18
Diabetes mellitus	12		14.6		15		18.5		0.50
Dyslipidemia	42		51.2		46		56.8		0.48
Cardiovascular disease	9		11		12		14.8		0.46
Arrhythmias	7		8.5		8		9.9		0.77
Cancer	10		12.2		7		8.6		0.46
COPD	10		12.2		15		18.5		0.26
Hypothyroidism	25		30.5		21		25.9		0.52
Gastroesophageal reflux	10		12.2		17		21		0.13
Depression/anxiety	33		40.2		36		44.4		0.59
Epilepsy	2		2.4		0		0		0.16
Parkinson’s	5		6.1		0		0		0.02
Migraine or headache	8		9.8		7		8.6		0.81
Pain/bruxism/ fibromyalgia	18		22		31		38.3		0.02

**Table 2:** Chronic diseases before and after receiving high doses of melatonin.

Disease	Cases Before (*n*)	Cases After (*n*)	b (Improve)	c (Worsen)	χ^2^	*p*
Arterial hypertension	54	9	45	0	45	<0.001
Ischemic heart disease	6	0	6	0	6	≈0.01
Diabetes mellitus	12	5	7	0	7	<0.01
Cancer	4	6	0	2	2	≈0.15

**Table 3:** Comparative Analysis of Chemical Laboratory Variables in Melatonin and Control Groups.

Variable	Normal Values	Melatonin				Control				*p*
		N = 81				N = 81				
		Min	Max	Mean	SD	Min	Max	Mean	SD	
Age (years)		55	98	74.4	9.1	52	92	73.9	8.9	0.71
GOT	10–42	9	39	17.9	4.70	7	37	16.9	4.3	0.20
GPT	10–40	6	58	16.5	8.63	5	42	15.4	6.5	0.37
ALP	31–100	16	101	64.6	17.03	34	325	75.0	34.4	0.02
Total bilirubin	0.10–1.40	0.10	1.38	0.62	0.24	0.29	1.56	0.61	0.2	0.93
Direct bilirubin	0.00–0.40	0.09	0.90	0.15	0.12	0.09	0.29	0.12	0.1	0.09
Total cholesterol	<200–239	42	290	182.9	50.2	99	295	181.5	39.5	0.84
Triglycerides	<150–199	35	358	119.9	55.8	47	269	120.9	49.6	0.91
HDL cholesterol	≥40	27	125	57.1	15.8	28	86	55.3	12.6	0.44
LDL cholesterol	<110–129	25	360	115.5	49.3	49	210	103.9	33.4	0.10
25-hydroxyvitamin D	>30	9.2	63.8	33.4	11.8	11.7	58	32.8	11.4	0.80
Ferritin	10–204	12.3	795	166.6	142.2	0	489.6	141.3	113.5	0.35
Transferrin saturation	20–50	8.0	75	31.4	11.4	0	82.0	29.1	17.2	0.44
Vitamin B12	187–883	120	2000	521.4	427.8	0	2000	488.8	417.9	0.69
Vitamin B6	Female: 2.0–32.8; Male: 5.3–46.7	5.2	76.6	24.3	16.5	0	92.6	23.9	25.0	0.96
Total leukocytes	4000–10,000/mm^3^	4187	61,300	7448	6372	142	14,766	7104	1969	0.65
Platelets	150,000–450,000/mm^3^	143,200	2,752,000	318,377	369,586	37,300	380,900	244,677	64,278	0.09

Mann–Whitney tests were used to compare differences between groups. GOT (glutamic oxaloacetic transaminase): IU/L, kinetic UV method; GPT (glutamic pyruvic transaminase): IU/L, kinetic UV method; ALP (Alkaline Phosphatase): IU/L, kinetic UV method; total bilirubin, mg/dL, diazo method; direct bilirubin: mg/dL, diazo method; total cholesterol, mg/dL, enzymatic method; triglycerides; mg/dL, enzymatic method; HDL (High-Density Lipoprotein) cholesterol: mg/dL, homogeneous system method; LDL (Low-Density Lipoprotein) cholesterol: mg/dL, calculated method; 25-hydroxyvitamin D: ng/mL, ELISA; ferritin: ng/mL, ELISA; transferrin saturation: % total iron-binding capacity; vitamin B12: pg/mL, ELISA; vitamin B6: ng/mL, LC-MS.

## Data Availability

The original contributions presented in this study are included in the article. Further inquiries can be directed to the corresponding author.
